# Supplementary material for: Vertically integrated spiking cone photoreceptor arrays for color perception
Source: Nat Commun. 2023 Jun 10;14:3444. doi: 10.1038/s41467-023-39143-8 (PMC10257685; doi:10.1038/s41467-023-39143-8)
Supplement: Supplementary file 1 — Supplementary information [file 41467_2023_39143_MOESM1_ESM.pdf]

Supplementary Information

Vertically Integrated Spiking Cone Photoreceptor Arrays for Color Perception

Xiangjing Wang[1], *Chunsheng Chen*[1], *Li Zhu*[3], *Kailu Shi*[1], *Baocheng Peng*[1], *Yixin Zhu*[1], *Huiwu Mao*[1], *Haotian Long*[1], *Shuo Ke*[1], *Chuanyu Fu*[1], *Ying Zhu*[1], *Changjin Wan*[1]*, *and Qing Wan*[1,2]*

[1] School of Electronic Science and Engineering, Collaborative Innovation Center of Advanced Microstructures, Nanjing University, Nanjing 210093, China.

[2] School of Micro Nanoelectronics, Zhejiang University, ZJU-Hangzhou Global Scientific and Technological Innovation Centre, 310027 Hangzhou, PR China.

[3] College of Integrated Circuit Science and Engineering, Nanjing University of Posts and Telecommunications, Nanjing 210003, China.

*Corresponding author:
E-mail: cjwan@nju.edu.cn and wanqing@nju.edu.cn

This PDF file includes:

Supplementary Fig. 1 Electrical characteristics of Ta_2O_5-based threshold switching memristor (TSM) with different sputtering times.

Supplementary Fig. 2 The negative differential resistance (NDR) performance of the TSM.

Supplementary Fig. 3 The charging and discharging process of the spike-encoder.

Supplementary Fig. 4 The output results of the spike-encoder at different current bias.

Supplementary Fig. 5 The absorption spectrum of the IGZO films.

Supplementary Fig. 6 Photoresistance of IGZO devices with different sputtering times.

Supplementary Fig. 7 The layer-by-layer structure of the vertically integrated spiking cone photoreceptor (VISCP) and the equivalent circuits.

Supplementary Fig. 8 X-ray photoelectron spectroscopy (XPS) scanning spectra of IGZO and Ta_2O_5 films.

Supplementary Fig. 9 The Atomic force microscope (AFM) diagram of IGZO and Ta_2O_5 films.

Supplementary Fig. 10 Device-to-device statistics of switching voltage and spike frequency.

Supplementary Fig. 11 The color-blindness test style images (CBTS-images).

Supplementary Fig. 12 The generation procedure of the training/test set.

Supplementary Fig. 13 Binarized mapping images of CBTS-images by VISCP devices with color vision.

Supplementary Fig. 14 Binarized mapping images of CBTS-images by VISCP devices with color-blindness vision.

Supplementary Fig. 15 Structure of the CNN for CBTS-MNIST recognition.

Supplementary Note 1 The details of the VISCP design.

Supplementary Note 2 Generation of color-blindness test style MNIST written digits.

Supplementary Note 3 The generation procedure of training/test set for recognition tasks.

Supplementary Note 4 Simulation details of color and color-blindness recognition.

Supplementary Note 5 Power consumption estimation.

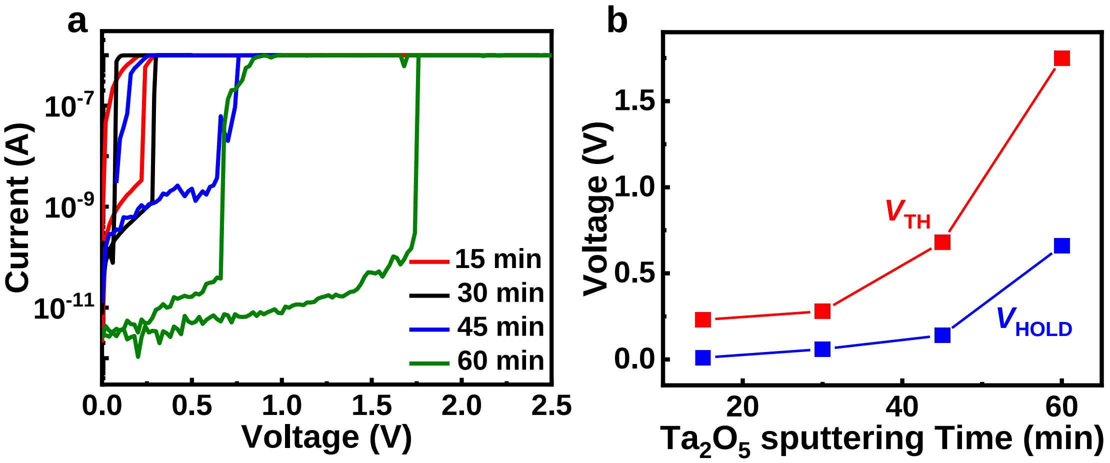

Supplementary Fig. 1 a, Current-voltage curves of the Ta_2O_5-based threshold switching memristor (TSM) of different sputtering times. **b,** The switching voltage distribution of the TSM with the different sputtering times.

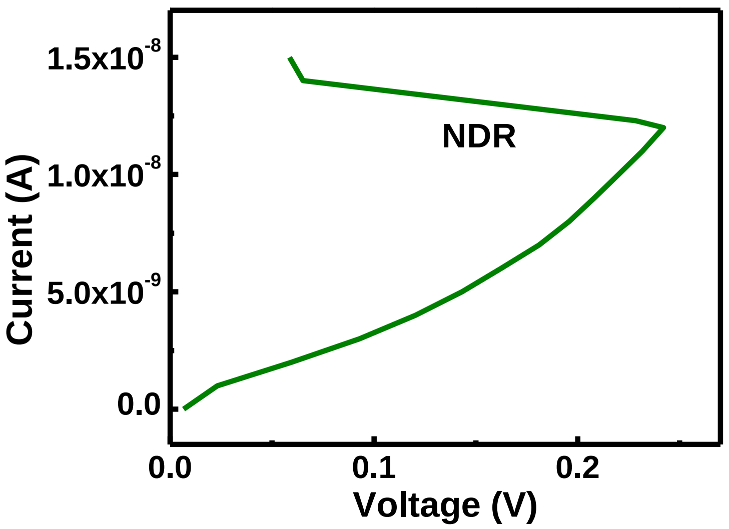

Supplementary Fig. 2 The negative differential resistance (NDR) behavior of the threshold switching memristor (TSM) under current-sweeping mode. The applied sweep current is from 0 to 15 nA in steps of 1 nA.

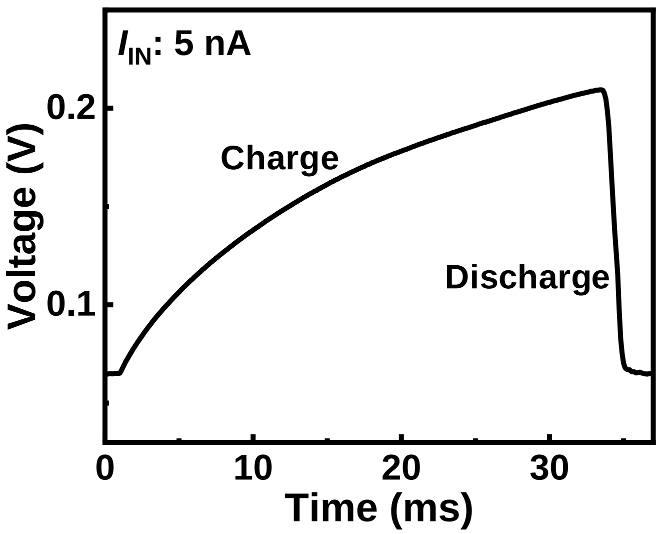

Supplementary Fig. 3 A single spiking characteristic of the spike-encoder. A fixed current of 5 nA is applied, and the process of charging and discharging can be observed clearly.

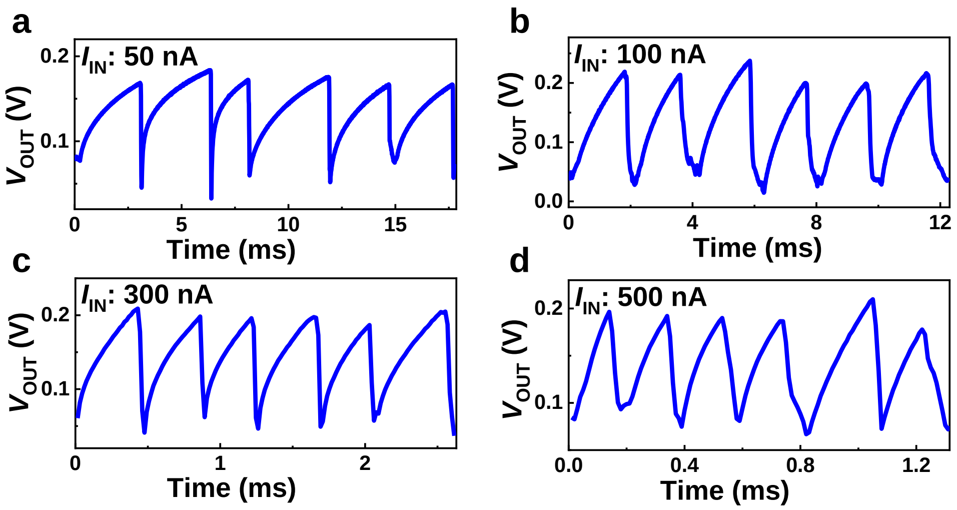

Supplementary Fig. 4 a-d, The outputs of the spike-encoder under the current bias of 50, 100, 300, and 500 nA, respectively.

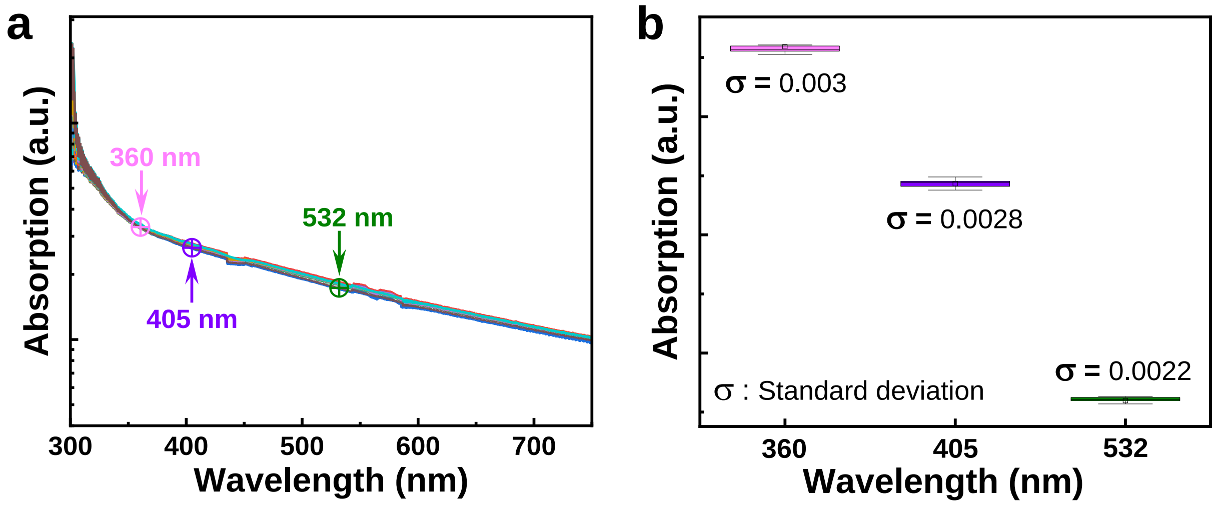

Supplementary Fig. 5 a, Absorption spectrum of the IGZO film. **b,** Device-to-device variations of light absorption with wavelengths of 360, 405, and 532 nm, respectively. The absorption spectrum were obtained from 20 samples which were deposited under the same deposition conditions.

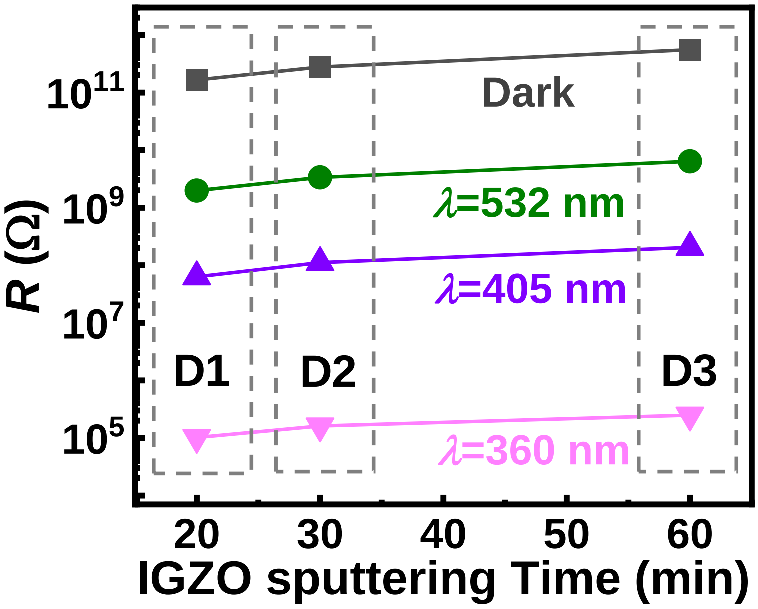

Supplementary Fig. 6 The resistance of IGZO devices 1-3 (D1-D3) with different sputtering times. The light intensity was kept constant at 0.5 nW/μm^2.

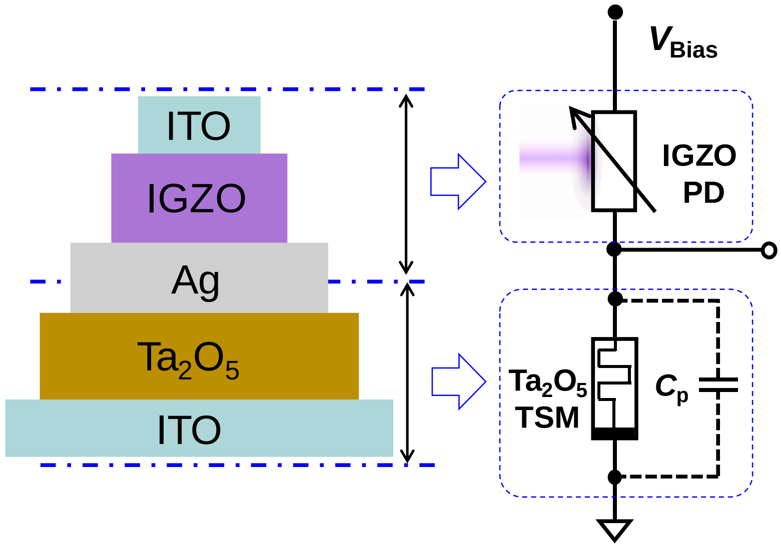

Supplementary Fig. 7 The layer-by-layer structure of the proposed device and the equivalent circuits for each component. The details of the vertically integrated spiking cone photoreceptor (VISCP) design can be found in Supplementary Note 1.

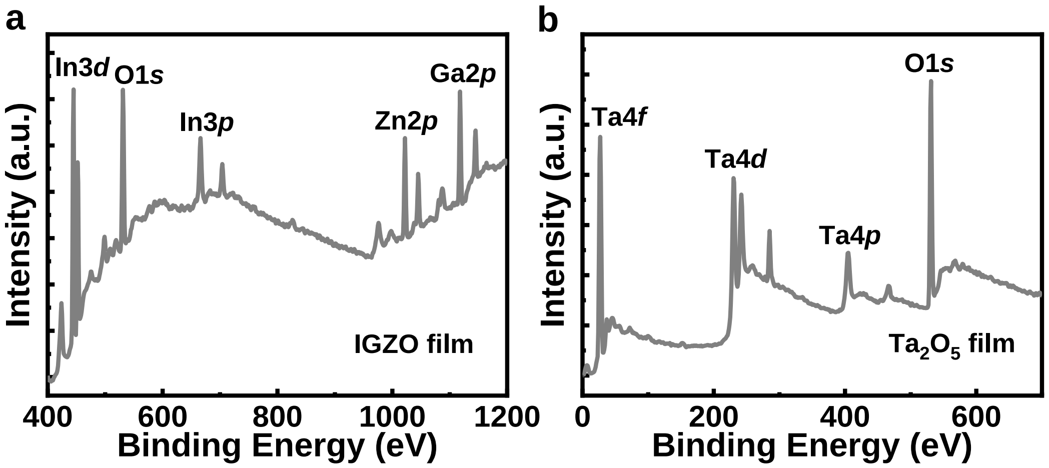

Supplementary Fig. 8 a, X-ray photoelectron spectroscopy (XPS) spectra of IGZO film. **b,** XPS spectra of Ta_2O_5 film.

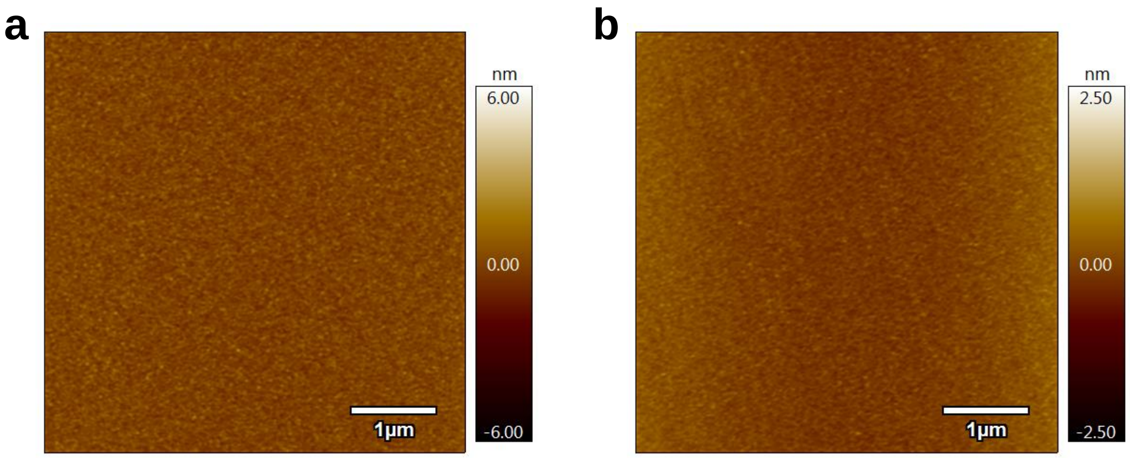

Supplementary Fig. 9 a, Atomic force microscope (AFM) diagram of IGZO film with a surface roughness of 0.4 nm. **b,** AFM diagram of Ta_2O_5 film with a roughness of 0.2 nm

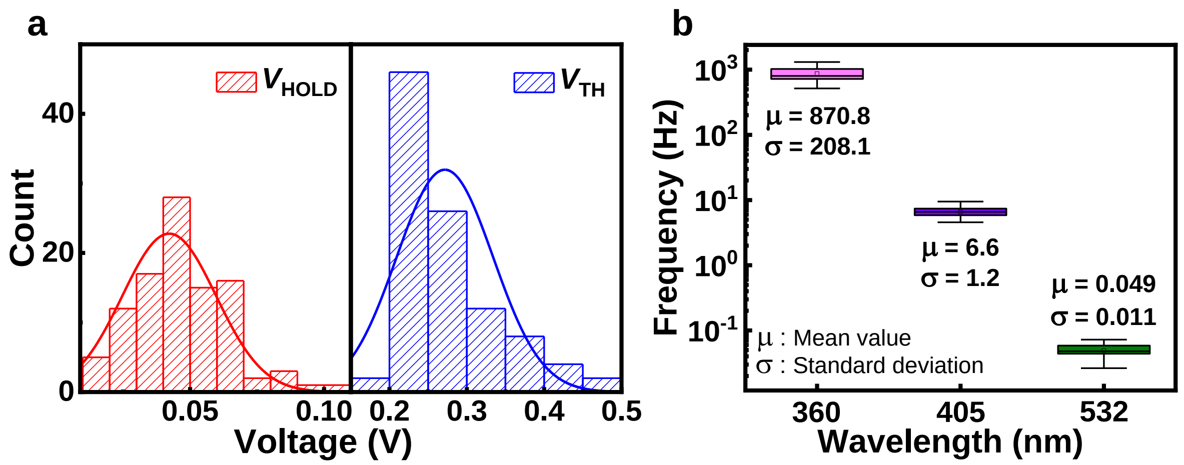

Supplementary Fig. 10 a, Device-to-device distributions of switching voltages extracted from 100 randomly selected devices. **b,** Spike frequency statistics at three illumination wavelengths. The light intensity was kept constant at 0.5 nW/μm^2.

96 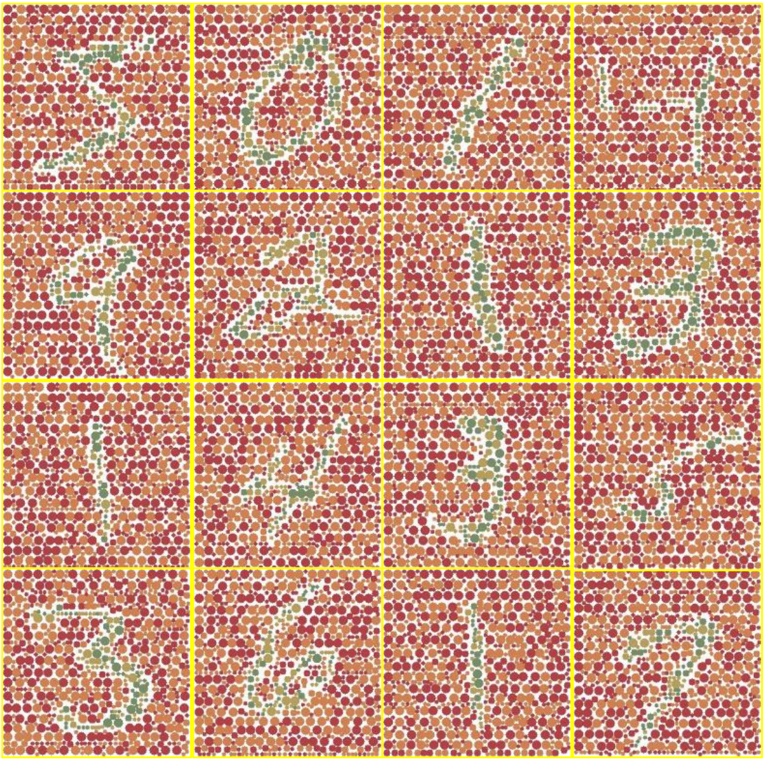

97 **Supplementary Fig. 11** The no. 1-16 images of color-blindness test style (CBTS-image). The

98 images are treated from MNIST and the labels are 3, 0, 1, 4, 9, 2, 1, 3, 1, 4, 3, 6, 3, 6, 1, 7,

99 respectively. The detailed processes can be found in Supplementary Note 2.

100

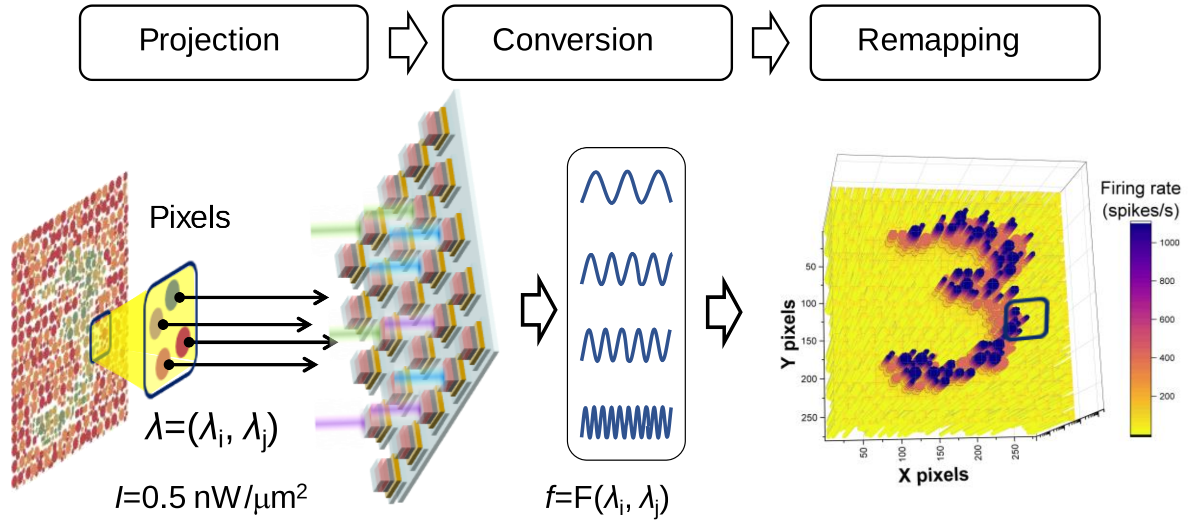

Supplementary Fig. 12 The generation procedure of the training/test set. The detailed processes can be found in Supplementary Note 3.

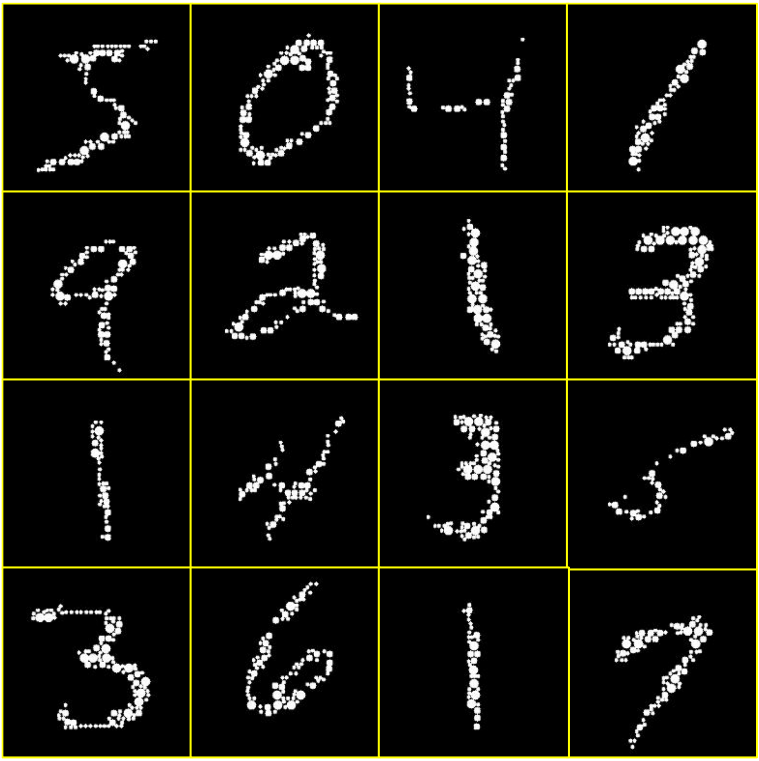

Supplementary Fig. 13 The mapping of no. 1-16 CBTS-images by VISCP devices with color vision were binarized with a threshold of 423 spike/s. The labels are 3, 0, 1, 4, 9, 2, 1, 3, 1, 4, 3, 6, 3, 6, 1, 7, respectively. Each pixel of a mapped image represents a spiking rate and is binarized by comparing with the threshold. The black one represents ‘0’, which means the spiking rate is lower than the threshold. The white one represents ‘1”, which means the spiking rate is not lower than the threshold.

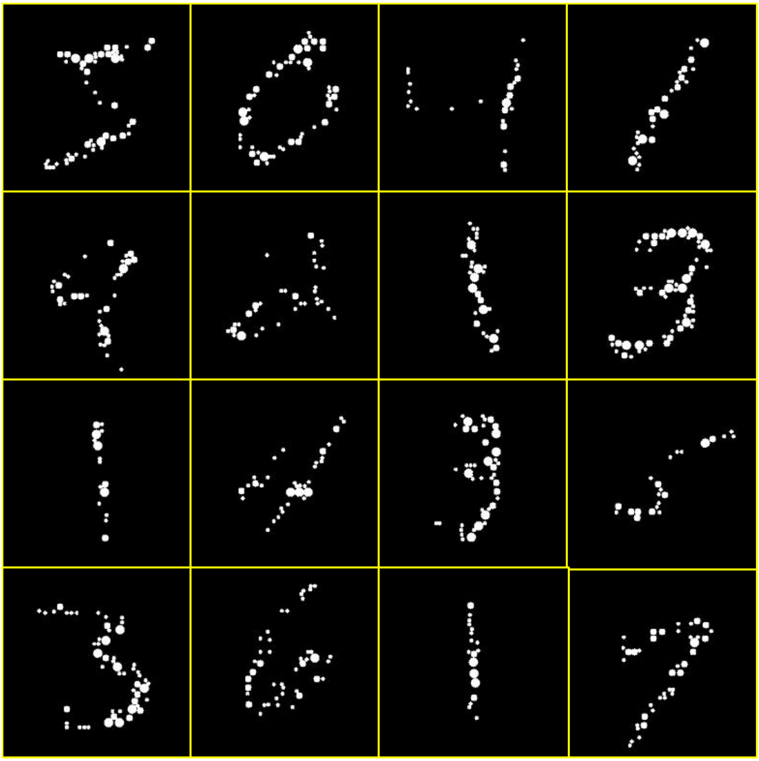

Supplementary Fig. 14 The mapping of no. 1-16 CBTS-images by VISCP devices with color-blindness vision were binarized with a threshold of 423 spike/s. The labels are 3, 0, 1, 4, 9, 2, 1, 3, 1, 4, 3, 6, 3, 6, 1, 7, respectively. Each pixel of a mapped image represents a spiking rate and is binarized by comparing with the threshold. The black one represents ‘0’, which means the spiking rate is lower than the threshold. The white one represents ‘1”, which means the spiking rate is not lower than the threshold.

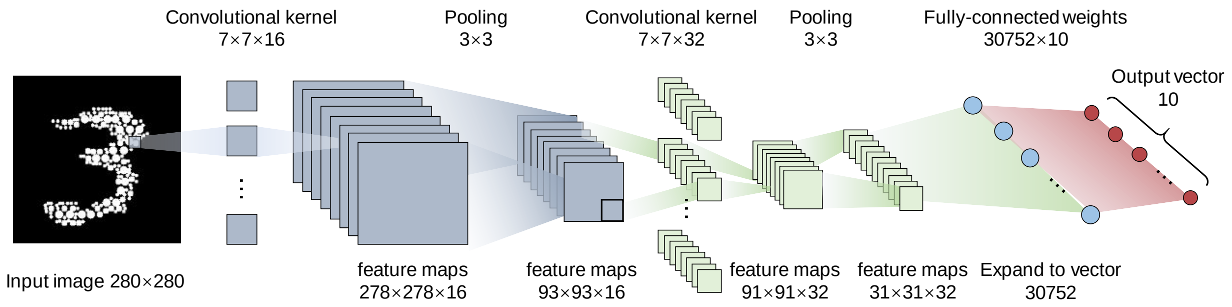

Supplementary Fig. 15 Structure of the five-layer CNN for CBTS-MNIST recognition.

Supplementary Note 1. The details of the VISCP design

The first layer from up to bottom is an indium oxide (ITO) film which is served as the transparent electrode. A voltage bias was applied in this electrode to obtain the spiking output. The input light can pass through the ITO electrode and penetrate into the second layer. The second layer is an indium-gallium-zinc-oxide (IGZO) film in which the conductance can be tuned depending on the intensity and wavelength of the light input. The third layer is a conducting layer of silver which is to connect the two functional devices (IGZO-based photoresistor and Ta_2O_5-based threshold switching memristor). This silver layer is also served as the output terminal where the output spikes can be measured. What's more, the layer can be served as Ag source enabling the formation of the Ag-based conducting path through the fourth layer. The fourth layer is the tantalum oxide (Ta_2O_5) which is served as the medium for silver migrants. The formation/rupture of a silver-based conducting path can occur in the Ta_2O_5 layer under a certain voltage, inducing a sudden change in conductance of the Ta_2O_5-based threshold switching memristor. The fifth layer is the ITO transparent electrode. This electrode is connected to the ground, which is also served as the substrate for the whole device. When V_{Bias} is applied and the TSM is in a high resistance state (HRS), the parasitic capacitor (C_{P}) begins to charge. When the C_{P} voltage exceeds V_{TH}, the TSM switches to a low resistance state (LRS). As a result, the capacitor is discharged through the on-state memristor. The voltage on the C_{P} then drops below V_{HOLD} and the TSM returns to HRS. Such a charging and discharging process forms a spike train with a certain frequency.

Supplementary Note 2. Color-blindness test style MNIST written digits

An image of color-blindness test style (CBTS-image) consists of circle-based patterns. In this work, the whole processes of generating CBTS-images can be simplified by two steps: find center points of circles with several numbers of radius and then paint each circle with specific colors.

Firstly, the origin MNIST written digits (the size of each image is 28×28) were enlarged 100 times (the size of each treated image is 280×280).

The second step is to find the center points for each circle pattern. One MNIST image is divided into two parts: the background and the digit. Center points were firstly searching within the background and then the digit part. Circles with three radiuses (r) of 3, 5, and 7, were generated as follows. Firstly, the center points of the circles with radius of 7 were generated by searching either the background or digit of a MNIST image. Then, the center points of the circles with radius of 5 and 3 were generated in sequence. To avoid the overlap between each circle, the center distances were randomly set to 2 to 3 folds of the radius. Only if the distances between one center point ($c_p|r=r_0$) to all the closest center points (c_{p1}, c_{p2}, ..., c_{pk}) are larger than r_0, such a center point can be recorded. Such processes were implemented 3 times to ensure that most areas of an MNIST was modified to the circle-based pattern.

The final step is to paint all these generated circles. In this work, the RGB values for red, orange, olive, and green are set to (209, 129, 81), (178, 65, 69), (120, 140, 103), (184, 158, 93), respectively. The circles in the background were randomly painted to red or orange, and circles in the digit part were randomly painted to green or olive.

Supplementary Note 3. The generation procedure of training/test set for recognition tasks

The generation process of training set for color-vision and color-blindness recognitions is shown in Supplementary Fig. 12. For color vision, the device can selectively respond to the four ‘colors’ (i.e., red, orange, olive, green) with four spiking rates of 0.2, 3.57, 423, and 1100 spike/s, respectively, which are extracted from Fig. 4b of the main text. For color-blindness vision, the device ‘cannot’ discriminate the orange and olive, and the output spiking rates are 3.57 spike/s for both cases. Therefore, the MINST-based CBTS-image (with RGB pixels) can be mapped to an image with each pixel of spiking rate.

The mapped images were then binarized and a threshold of 423 spike/s was set. In this case, only the spiking rate of a pixel less than this value is set to 0, otherwise, the pixel is set to 1. The binarized results for color vision and color-blindness vision are shown in Supplementary Figs. 13 and 14, respectively.

Supplementary Note 4. Simulation details of color and color-blindness recognition

In this work, 5000 binarized images were used for recognition tasks. The 4500 images were used as the training dataset and the others were used as the test dataset. A five-layer convolutional neural network (CNN) was used (Supplementary Fig. 15). The first layer consists of an input image with dimensions of 280×280. The input data is then convolved with 16 filters of 7×7 in size and padding of 2, resulting in dimension of 278×278×16. The second layer is a Pooling operation with filter size of 3×3. Hence the resulting image dimension is 93×93×16. Similarly, the third layer also involves in a convolution operation with 32 filters of 7×7 in size and padding of 2 followed by a fourth pooling layer with same filter size of 3×3. The resulting image dimension is then reduced to 31×31×32 in size. The fifth layer is a fully connected layer with 10 nodes to identify the label of each treated digits. In this layer, each of the 10 nodes will be connected to the 30752 (31×31×32) nodes from the previous layers. The recognition consists of two phases: training and inferencing. The training phase includes two sub-procedures: forward-pass and weight update. The weight of each node is updated based on back propagation algorithm. During the inferencing phase, the network was fed up with the test images to identify the label of them, and the recognition accuracy is thus obtained.

Supplementary Note 5. Power consumption estimation

By configuring the IGZO-based photoresistor, the output of the Ta_2O_5-based spike-encoder could be steadily obtained. The input voltage is set to 0.5 V. The IGZO's resistance (R_I) remains constant at a specific light wavelength and intensity. A spike consists of two stages, charging and discharging, and T_{rise} and T_{fall} are defined as the charging time and discharging time. The encoder remains in the high resistance state (R_{OFF}) during charging and in the low resistance state (R_{ON}) during discharging, so the power consumption per spiking of the whole circuit is calculated to be:

$$P_{spike} = \frac{1}{T}\int_0^T \frac{V_{IN}^2}{R}\mathrm{d}t = \frac{1}{T}\left(\frac{V_{IN}^2}{R_I+R_{OFF}}T_{rise} + \frac{V_{IN}^2}{R_I+R_{ON}}T_{fall}\right) \quad (1)$$

It can be seen from the equation that the power consumption increases with the decrease of R_I. The power consumption is less than 41 pW under 532 nm light illumination. The power consumption is less than 400 pW under 405 nm light illumination. The power consumption of VISCP is extremely similar to that of the biological cone under visible light illumination (~200 pW). The power consumption is less than 210 nW under 360 nm light illumination.
